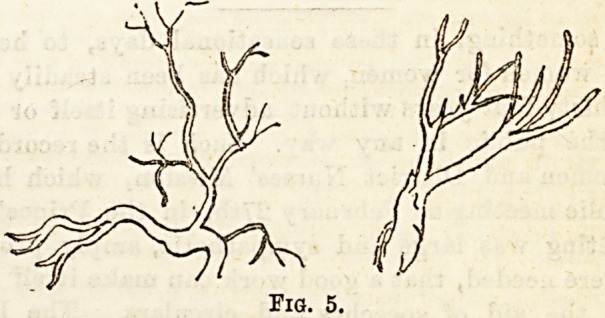# The Microscope in Medicine

**Published:** 1891-03-07

**Authors:** Frank J. Wethered


### The Microscope in Medicine.

By Frank J. Wethered, M.D.

### VIII.—THE EXAMINATION OF SPUTUM.

By "Sputum" is meant all the matter coughed or hawked up from the air passages. Since this material includes the secretions from the buccal-respiratory tract it might naturally be expected that an examination of them would yield valuable results as regards the condition of these parts; and such is actually the case. Since the discovery of the tubercle bacillus by Koch, in 1882, the microscopic examination of sputum has received a great impetus, and it has now become almost a matter of routine in any case of lung disease of a doubtful character. As our knowledge of pathological chemistry increases, the importance of accurate and regular investigation of the contents of the spitoon will become more and more important, and hence it behoves every practitioner to make himself acquainted with the more important processes which are employed. Not only by the use of stains can information be derived, but also unstained specimens are of great aid in establishing a diagnosis.

In these articles an attempt will be made to describe briefly the methods of examination in everyday use.

Too much importance cannot be laid on the collection of the sample. Great annoyance and loss of time is caused by the admixture of food stuffs with the expectoration, and care, therefore, must be exercised to see that the spitoon is perfectly clean, and also to request the patient not to make it a receptacle for grape-skins, orange-pips, etc.; and if the whole of the secretion for twenty-fours be required to use a fresh one during meals.

For nearly all purposes, however, it is preferable to select that portion which has been ejected on first waking in the morning before any food or drink has been taken. Any particles of milk are particularly apt to mislead, and so are minute pieces of bread or starchy food.

The specimen is thrown into a flat dish, preferably a black vulcanite one. A small portion is then removed to a glass-slide and a cover-glass laid on it, and pressed out by gentle pressure. The parts to select are the most opaque portions, and only sufficient should be taken to form a uniform layer under the glass. The removal is most easily accomplished by means of scissors or forceps, or by two steel pens, fixed in holders, which thus act both as teazers and lifters.

A quarter-inch-lens should be employed, and a diaphragm with the smallest aperture.

The different objects to be met with will now be described *seriatim*:

Epithelium.—Three varieties may be noticed, squamous, columnar, and cubical.

*Squamous.* (Fig. 1, *a*).—This is derived from the mouth, pharynx, and upper part of the larynx. The cells are met with in all sputa in variable numbers, sometimes in huge quantities, packed together, giving an appearance, if turned edge-way, of crystals or elastic tissue, this error may be corrected by gently moving the cover-glass, so causing them to turn over.

Fig. 1.

The cells are often of a rather irregular shape, and may then be mistaken for "cancer cells"; whether such are ever found in the expectoration is questionable, but if they were they would probably be accompanied by blood-cells and other signs of ulceration.

The diagnostic significance of the presence of these cells is, of course, very little.

*Columnar Cells* (Fig. 1. *b*).—These are derived from the trachea and other respiratory passages. If some of them are possessed of cilia, they are probably derived from the nasal passages rather than from the surface of the trachea. If these cells are present in large numbers they indicate a catarrh of parts from which they came.

*Cubical Cells* (Fig. 1. *c—Alveolar Epithilum*).—The occurrence of these cells is of far more importance; if present in any number they are indicative of an inflammatory process going on in the air cells, such as tuberculosis or pneumonia.

Each cell possesses a nucleus. The cells are finely granular, and frequently contain pigment granules, or particles of carbon. They are seen in all stages of fatty degeneration.

*Leucocytes, pus, and mucus Cells* (Fig. 1. *d*).—These are constant ingredient of all sputa; they can hardly be distinguished from each other. The leucocytes are often seen to be undergoing fatty degeneration, and to contain particles of carbon, &c. In cases of purulent bronchitis or when an empyema has burst into the lungs, the expectoration may consist almost entirely of pus cells.

Other bodies similar to these are "*salivary corpuscles.*" These are larger and more coarsely granular than the white blood corpuscles.

*Red Blood Cells* (Fig. 1. *e*).—These occur in the expectoration in variable numbers. In cases of hæmoptysis, of course, scarcely anything else can be seen, but the consideration of this subject is of more interest clinically than from the point of view under which we are now considering it.

The red corpuscles can be easily recognised by their uniform circular appearance and their tendency to run together into rouleaux. Some information as to their source may be obtained by observing the epithelium, elastic tissue, &c., which accompany them.

When blood has remained for some time in the air passages the corpuscles may be broken up, and crystals of *Hœmatin* will then often appear in the expectoration (Fig. 1.*f.*)

FIG. 2.

FIG. 4.

*Elastic Tissue* (Fig. 2).—The search for the "curled fibres" is second only in importance to that for tubercle bacilli.

The expectoration for twenty four hours should be collected and thrown on to a black vulcanite dish, and any small opaque particles should be picked out with forceps and placed on a slide. This usually suffices to find the fibres should they be present in the sputum, but, if further investigation be desired, the solvent method introduced by Dr. Fenwick should be adopted.

The expectoration is boiled for a few minutes in a beaker with an equal quantity of a solution of caustic soda, 20 grains to the ounce. The mixture is occasionally stirred with a glass rod, and until the fluid is rendered diffluent more soda must be added, if necessary. The beaker is then emptied into a large conical glass containing water, and the sediment allowed to settle, portions of which are removed to glass slides for examination.

Elastic fibres are easily recognised by their clear outline, by their branching and anastomosing so forming a rough cast of the alveoli, their yellow colour and homogeneity, and their indestructibility.

FIG. 3.

Complete loops, however, are not often seen; small fragments are more common, and the acuteness of the destructive process may to some extent be measured by their "tailed" or alveolar arrangement. This tissue may be derived not only from the parenchyma of the lung, but also from the upper part of the respiratory tract. When the latter is their origin, the arrangement of the fibres is much more linear.

Their presence does not necessarily indicate a tubercular process taking place in the respiratory organs, but destruc-of lung tissue, either in phthisis, bronchiectasis, or pulmonary abscess.

Some interesting objects are found in the sputa of asthmatic patients, especially if this be collected during a paroxysm; the most important of these are Curschmann's Spirals and the Charcot-Leyden Crystals.

*Curschmann's Spirals* (Fig. 3).—To observe these the sputum is examined in the ordinary way, the most solid-looking particles being selected for examination. The spirals occur in three forms. The most complete consists of a highly refracting "central thread" round which is entwined an en-sheathing layer of mucoid material, the whole having a peculiar spiral arrangement. Occasionally the central thread only is seen, whilst the third form consists of the coil of mucoid material only.

These bodies are not only found in asthma, but also in resolving pneumonia, and they seem to indicate a catarrh of the smallest bronchi.

*Charcot Leyden Crystals* (Fig 4).—These take the form of colourless pointed octahedra. They are supposed by Schreiner to consist of a phosphate of a new base. Their diagnostic value is small.

FIG. 5.

Another interesting set of bodies are the "bronchial stolons" (Fig 5). These are characteristic of "plastic bronchitis." The sputum in this disease consists of small pellets covered with mucus. If these be thrown into water they spread out and are then seen to consist of almost perfect casts of the bronchial tree. They are usually about one and a-half to two inches in length; the diameter is narrow—their colour is usually a yellowish white—they divide dichotomously. At the points of branching a slight bulging may be noticed.

Other objects very similar to these in appearance are the branching clots which have been formed in the air-passages as the result of hæmorrhage. These lack the laminated structure of the stolons.

Cholesterin and crystals of oxalate of lime and triple phosphates are occasionally found in the sputum, but are of little importance.

Attention must finally be drawn to fragments of hair, linen, &c., which often fall accidentally into the spitoon and may be mistaken for fragments of elastic tissue.

---

MARGARINE, as is generally known, can no longer be exposed for sale as butter or under any other name than its own. Notwithstanding this, it appears from the police reports that, "by mistake," margarine is occasionally supplied for butter to the unsuspecting victim. To send a basket of margarine in the place of a basket of butter is a curious "mistake" for a provision merchant to make. We do not hear of baskets of butter being delivered in mistake for margarine, such an error would be so very unprofitable!

## Figures and Tables

**Fig. 1. f1:**
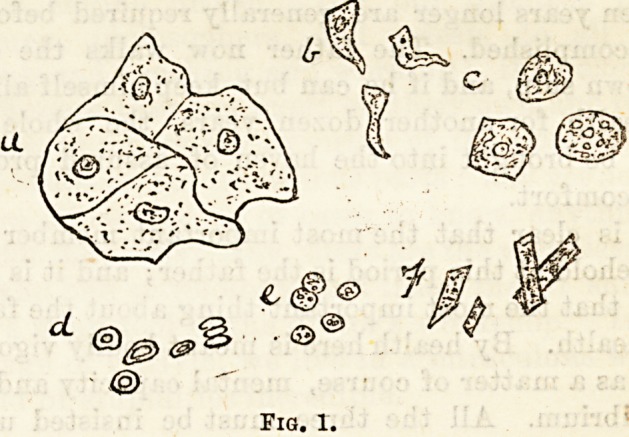


**Fig. 2. f2:**
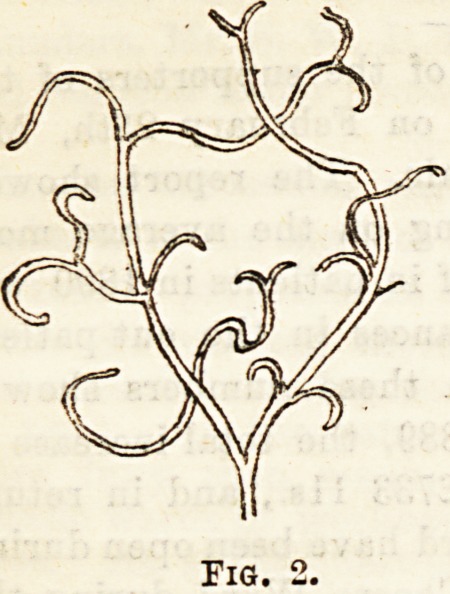


**Fig. 3. f3:**
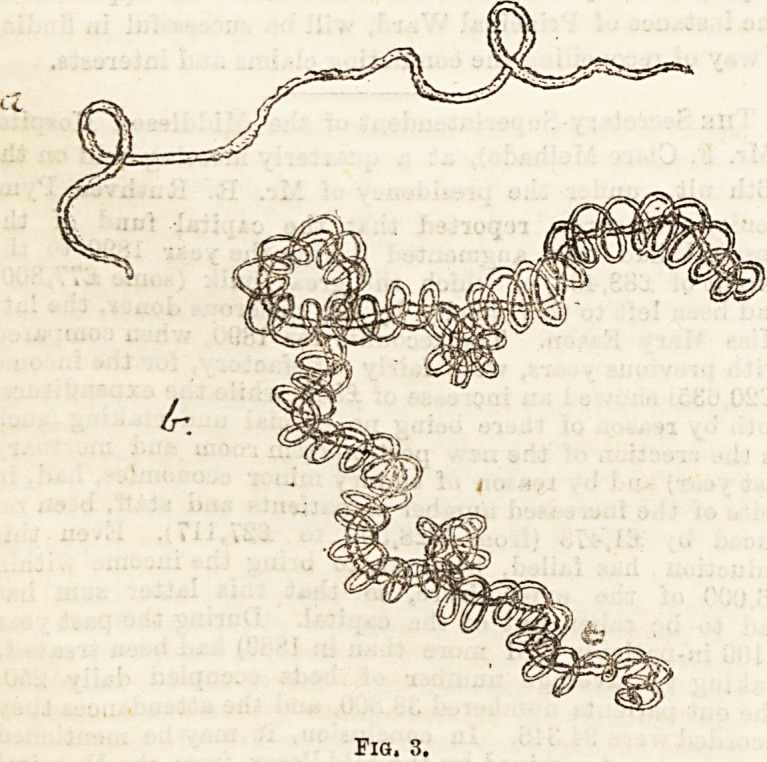


**Fig. 4. f4:**
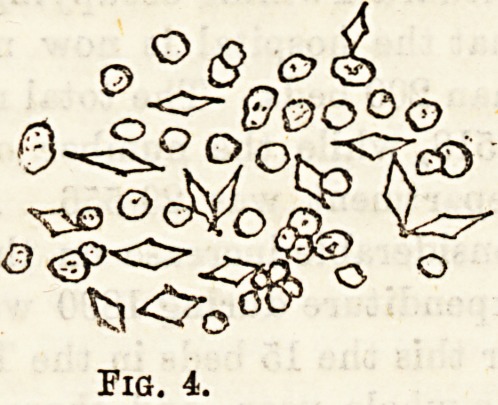


**Fig. 5. f5:**